# Spatial Variation as a Tool for Inferring Temporal Variation and Diagnosing Types of Mechanisms in Ecosystems

**DOI:** 10.1371/journal.pone.0089245

**Published:** 2014-02-20

**Authors:** Matthew P. Hammond, Jurek Kolasa

**Affiliations:** Department of Biology, McMaster University, Hamilton, Ontario, Canada; National University of Mongolia, Mongolia

## Abstract

Ecological processes, like the rise and fall of populations, leave an imprint of their dynamics as a pattern in space. Mining this spatial record for insight into temporal change underlies many applications, including using spatial snapshots to infer trends in communities, rates of species spread across boundaries, likelihood of chaotic dynamics, and proximity to regime shifts. However, these approaches rely on an inherent but undefined link between spatial and temporal variation. We present a quantitative link between a variable’s spatial and temporal variation based on established variance-partitioning techniques, and test it for predictive and diagnostic applications. A strong link existed between spatial and regional temporal variation (estimated as Coefficients of Variation or CV’s) in 136 variables from three aquatic ecosystems. This association suggests a basis for substituting one for the other, either quantitatively or qualitatively, when long time series are lacking. We further show that weak substitution of temporal for spatial CV results from distortion by specific spatiotemporal patterns (e.g., inter-patch synchrony). Where spatial and temporal CV’s do not match, we pinpoint the spatiotemporal causes of deviation in the dynamics of variables and suggest ways that may control for them. In turn, we demonstrate the use of this framework for describing spatiotemporal patterns in multiple ecosystem variables and attributing them to types of mechanisms. Linking spatial and temporal variability makes quantitative the hitherto inexact practice of space-for-time substitution and may thus point to new opportunities for navigating the complex variation of ecosystems.

## Introduction

The spatial texture of a landscape is a fundamental reflection of the ecological processes underpinning it. Thus, spatial distributions of population [1], geological [2] and climatological variables [3] can impart key details about the forces, operating over time, that forged them. Spatial patterns are *diagnostic* when they are used to uncover hidden mechanisms in the landscape, and *predictive* when they indicate the likely future behavior of a process. Ecology is full of examples of the former, diagnostic approach where spatial patterns are mined for evidence of mechanisms like dispersal, competition or environmental structuring [4]–[7]. But the latter, predictive approach is also commonplace. Because obtaining long time series is difficult, inferring temporal patterns from spatial data is used in such varied contexts as: (i) chronosequences, where gradients of different-aged sites are used to track how a process (e.g., succession) changes from one state to another over time [8]–[11], (ii) boundary dynamics, where spatial snapshots can reveal the rate of species spread [12], (iii) complex dynamics, where spatial data helps detect chaos [13], and (iv) regime or phase shifts, where changes in spatial variation can expose the incipient reorganization of an ecosystem [14]–[16].

Using spatial patterns to infer temporal patterns (“space-for-time substitution”; *sensu* [8]) or mechanisms quickly encounters the hard problem of interpreting dynamics [17], [18]. We, as others [18], [19], note that progress will require a deep understanding of what spatial patterns reveal about temporal patterns, and how these come together in the spatiotemporal patterns of landscapes. We further suggest that such insight will help both *predictive* and *diagnostic* efforts.

Here we focus on the variability of values over time (i.e., the inverse of stability [20] or constancy [21]) as opposed to properties like resistance or resilience [21]. We focus, therefore, on the dissimilarity of values and whether this variability (e.g., fluctuations in density) can be predicted from a snapshot of spatial variability. This application, in particular, would be useful given the rarity of long time series in ecology [9], the widespread nature of anthropogenic impacts [22], the increasing attention to ecological stability [20], [23]–[26], and the need for clearer links between spatial and temporal concepts [27].

**Figure 1:**
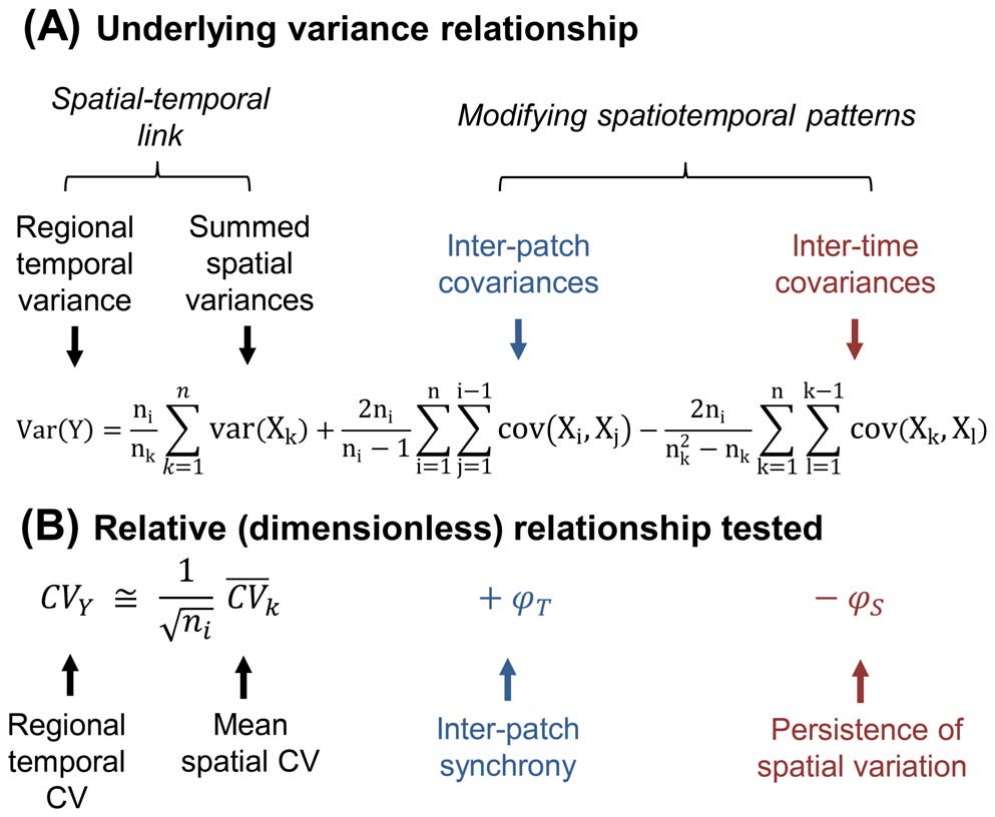
The spatial-temporal variability link. (A) We derived an analytical relationship linking regional temporal variance of a process (Var(Y)) to summed spatial variances at time *k* (∑var(X_k_)). Inter-patch synchrony (∑cov(X*_i_,*X*_j_*)) and persistence (∑cov(X*_k_,*X*_l_*)) modify this link and lead temporal and spatial variance to scale as a function of number of patches (*n_i_*) and time points (*n_k_*) when these terms are zero. (**B**) We evaluate the usefulness, for prediction and description, of the corresponding (relative) relationship that uses dimensionless coefficients: Regional temporal CV (CV_Y_), mean spatial CV (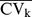), and indices of synchrony (φ_T_) and persistence (φ_S_). While an exact solution exists (Eq. S28, File S1), we use a more useful approximation, 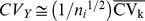, that gives an expected temporal CV for a given spatial CV when synchrony and persistence are negligible (Eq. S31,File S1).

**Figure 2:**
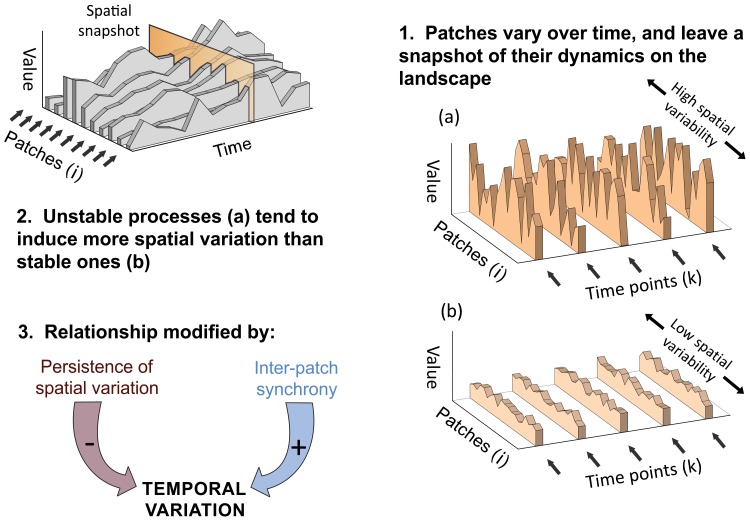
Spatial imprinting of ecosystem processes. Theorized mechanism by which temporal fluctuations of patches create spatial variability in the landscape, which may in turn be a proxy for temporal variability. Spatiotemporal patterns (inter-patch synchrony and persistence) modify the correspondence of spatial and temporal variability (Fig. 1), so it is unknown whether the link is strong enough for predictive (e.g., space-for-time substitution) applications and whether modifying terms have diagnostic/descriptive value.

To our knowledge, no links explicitly tie the temporal and spatial variation of a variable. However, a theoretical foundation for doing so is available through ANOVA variance partitioning [28]–[30] because overall spatiotemporal variation can be broken down into its spatial and temporal components. Crucially, these components can be re-expressed in terms of temporal variance at the regional scale (i.e., spatially-aggregated at time *k*; given as Var(Y) where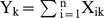). This scale reflects the net sum of what occurs in all patches and thus reflects variation in resources and ecological functions at the landscape level. Our derivation makes regional temporal variance itself decomposable (Fig. 1A). Traditional variance partitioning methods, in contrast, only decompose total spatiotemporal variance. We show in Fig. 1A that regional temporal variance (Var(Y)) obeys a simple relationship with a spatial quantity - the sum of spatial variances measured at time *k* (∑var(X_k_)). This relationship, in turn, is modified by two spatiotemporal patterns [28]–[31], *inter-patch synchrony* and *persistence of spatial variation* (Figs. 1 & 2; see File S1 for derivation and details).

*Inter-patch synchrony* (summed inter-patch covariances; ∑cov(X*_i_,*X*_j_*)) refers to temporal changes that happen simultaneously in patches *i* and *j*. It is well known to boost temporal variation at the regional scale [32] (e.g., widespread population decline during drought). On the other hand, *persistence of spatial variation* (summed inter-time covariances; ∑cov(X*_k_,*X*_l_*)) - *persistence* for short - describes differences or gradients between patches *i* and *j* that are retained from time *k* to *l* (e.g., fixed or permanent differences between locations). Opposite in sign to synchrony, persistence is associated with lower temporal variance. This is because a pattern of spatial variation is retained over time only if most patches are relatively stable. Temporal variance thus depends critically on the balance of synchrony and persistence.

### Implications of Analytical Framework

The relationship captured in Fig. 1A points out the basis for *predictive* applications like space-for-time substitution. It does so by showing that spatial and temporal variance will scale exactly (by a factor of *n_i_*/*n_k_*) for stochastic processes. Stochastic processes enable this because their values are uncorrelated between patches *i* and *j*, as well as between times *k* and *l*, and this sets synchrony and persistence terms to zero (∑cov(X*_i_,*X*_j_*) = 0, ∑cov(X*_k_,*X*_l_*) = 0). This is a form of ergodicity [33] that can be illustrated by an analogy. Imagine a seascape in which wave peaks are independent of each other: In this null case, wave amplitudes from trough to peak would be equally large or small whether waves were measured from a fixed point (i.e., waves passing over time) or from a transect across the seascape (i.e., a snapshot of waves across space). Our formulation merely adds that this match between temporal and spatial variability applies at the regional (seascape) scale as well as at the patch (wave) scale. Fig. 2 summarizes this mechanism, showing how temporal fluctuations are recorded as spatial variability.

*Diagnosis*, where inferences are made about how patterns came about, may also be made possible by the analytical solution. This is because components of temporal variance from Fig. 1A also describe and summarize spatiotemporal patterns that are the net result of ecological mechanisms. Moreover, because these terms are linked to temporal variability, they may provide a new view of dynamics and their consequences for stability.

Because they are commonly used in ecology, we extended our analytical framework to include common indices (Fig. 1B) like the Coefficient of Variation (CV), and indices of synchrony (φ_T_) and persistence (φ_S_). We test the validity of these formulations and turn them to answering three questions: (i) Is the spatial CV of a landscape variable a meaningful proxy for its (regional) temporal CV? (ii) Under what conditions is it predictive? And (iii) what do departures from an exact match between spatial and temporal CV tell us about the forces shaping dynamics of variables? We apply our approach to 136 biotic and physicochemical variables from three landscape types: Laboratory arrays of connected aquatic microcosms (measured for 20 weeks), a natural array of Jamaican coastal rock pool ecosystems (13 years), and a set of seven lakes from the North Temperate Lakes LTER site (30 years). Results shed light on what real world inferences can be drawn when the relationship between spatial and temporal variation is known.

## Materials and Methods

### Ethics Statement

Invertebrate species were sampled with permission on land owned by University of West Indies (Discovery Bay Marine Lab) and are not protected by law. Laboratory experiments used invertebrate species that do not require permits or procedural approvals.

### Analytical Relationship: Linking Spatial and Regional Temporal Variance

Values in a landscape vary over time (*k…n*), and across patches (*i…n*). These dimensions of variation both contribute to regional temporal variance, which is the variance of the spatially-aggregated time series (i.e., Var(Y) where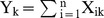). Spatial and temporal variation can be precisely linked through two mathematical truisms: (i) Spatial and temporal variances, estimated from the same site × time data matrix, are related by rules that underlie ANOVA variance partitioning and (ii) these variances, which capture variation at the aggregate scale for both time (i.e., Var(Y)) and for space (i.e., temporally-aggregated; Var(Z) where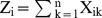), can be further decomposed into variances and covariances of patches *i* and *j* or time points *k* and *l* [34]. See File S1 for derivation. Var(Y) can thus be re-expressed as in Fig. 1A where; var(X_k_) is the spatial variance at time *k*, cov(X_i_,X_j_) is the covariance of patch *i* with *j* (synchrony), and cov(X_k_,X_l_) is the covariance of time *k* with *l* (persistence of spatial variation). These three components of spatiotemporal pattern are consistent with prior theory and statistical concepts [28]–[31].

**Figure 3:**
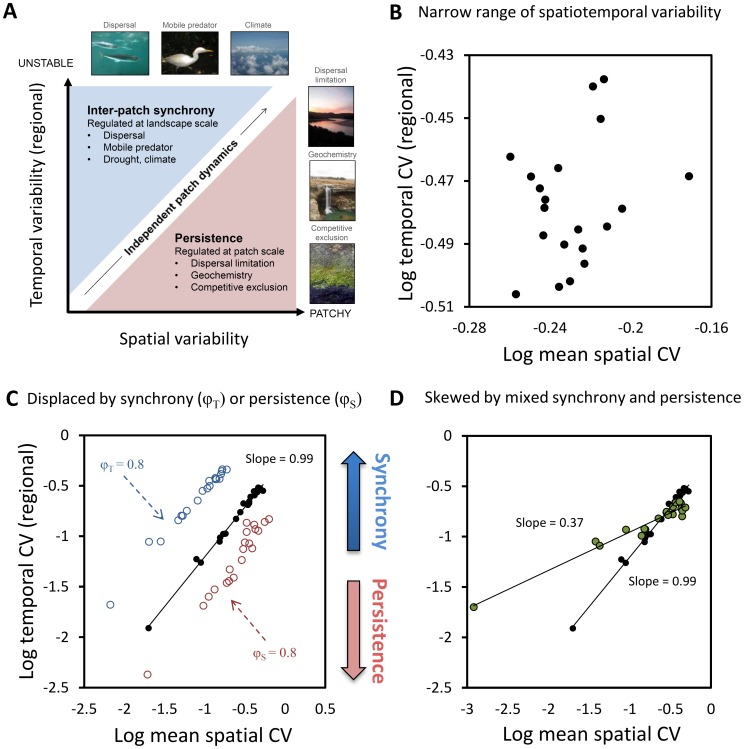
Anatomy of a plot between spatial and regional temporal variability. A stochastic null model of a three patch mosaic illustrated several features of a plot between log mean spatial CV and log regional temporal CV. (**A**) Three regions exist in which a variable (point) can fall - an “independent dynamics region” when values are independent between patches *i* and *j* and time points *k* and *l*, a “synchrony region” when inter-patch synchrony boosts temporal CV, and a “persistence region” when spatial gradients are retained over time; (**B**) Weak linear relationship when variables share similar spatiotemporal variability, leading to scatter from small variations in synchrony or persistence; (**C**) Strong linear relationships when variables differ in spatiotemporal variabililty and occupy the “independent dynamics region” (black circles), but also when all variables are equally dispaced by synchrony (blue circles) or by persistence (red circles); (**D**) Deviation of regression slope from ∼1 (black circles) when variables change in synchrony or persistence as a function of variability. Here, a gradient exists from variables with low variability and synchrony to variables with high variability and persistence. Spatial CV values are means of spatial CV measured at time point *k*. Each point represents a variable and is a mean of ten replicates.

**Figure 4:**
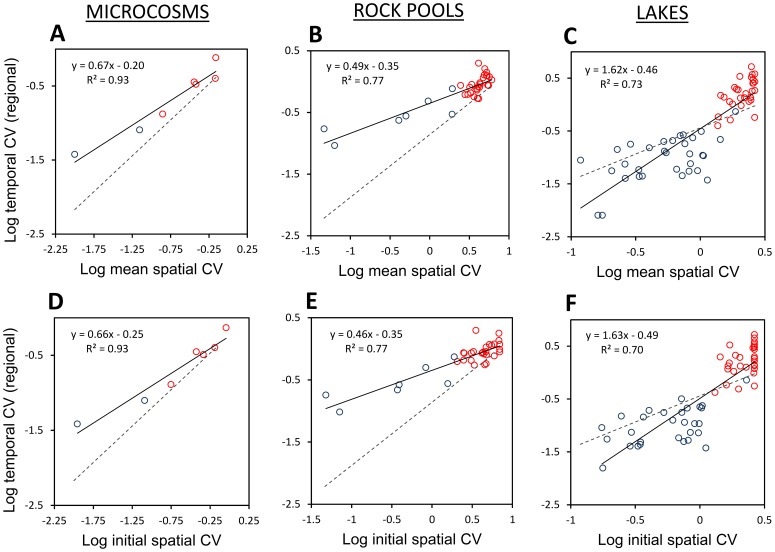
Empirical CV plots illustrating an underlying spatial-temporal link (Fig. 1B). The regional temporal CV of an ecosystem variable (data point) was predictable from its spatial CV in microcosm (n = 7) (**A, D**), rock pool (n = 33) (**B, E**), and lake systems (n = 60) (**C, F**). The predictive value of spatial variability was consistent in that linear associations emerged whether spatial variability was estimated as the mean of spatial CV’s at time *k* (**A–C**), or whether a spatial CV from an initial time point (*k*) was used to predict temporal CV of the remaining (*k+1…n*) time series (**D–F**). Dashed lines denote the relationship expected for stochastic processes i.e., when values are independent across space and time. These were obtained by simulating random numbers with the same data structure as empirical data sets. Abiotic variables (blue circles) were consistently more stable and less spatially patchy than biotic variables (red circles).

**Figure 5:**
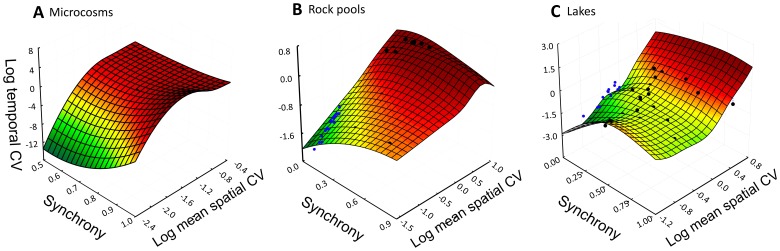
The modifying role of inter-patch synchrony. Relationship between spatial CV and regional temporal CV as modified by the degree of inter-patch synchrony in (**A**) microcosm, (**B**) rock pools and (**C**) lakes. Synchrony increased regional temporal CV relatively little over that explained by spatial CV. Black points = empirical variables, blue points = simulated, randomly-generated variables (n = 20; see File S1) to represent “independent dynamics region.”

We converted the above analytical relationship into dimensionless quantities (Eq. S28; File S1) – regional temporal CV (CV_Y_), spatial CV (CV_k_), and indices of synchrony (φ_T_) and persistence (φ_S_). While exact, this relationship does not yield a clear null relationship between CV_Y_ and spatial CV. We therefore used an approximation of it (Eq. S31; File S1) that gives the expected value of temporal CV from spatial CV in the absence of synchrony or persistence (Fig. 1B). Temporal CV values calculated using this approximation were 94–98% correlated (1∶1) with values from random number simulations where synchrony and persistence were close to zero. These null values, in turn, were used to plot the lines of “independent dynamics” shown in spatial-temporal CV plots (Figs. 3–5).

### Data Analysis

Biotic variables included population densities of invertebrate and fish species and ecosystem-level quantities like NPP, while physicochemical variables ranged from temperature and pH to ion concentrations (Table S1, S2). For each variable, we estimated all indices in Fig. 1B. Regional temporal CV was estimated as the quotient of the time series standard deviation and mean. Mean spatial CV of a variable was defined as the average of spatial CV’s measured at time *k*. These were calculated either across the three microcosms of each experimental replicate, across 49 Jamaican rock pools, or across the seven LTER lakes. If a species was absent from all water bodies spatial CV could not be calculated for that time point. In these cases, mean spatial CV was calculated as an average of the time points in which it was present (mean frequency of occurrence = 65% of years).

We estimated inter-patch synchrony using a variance ratio φ_T_ [35] which is, roughly speaking, a ratio of aggregate (regional) to component variances:
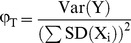
(1)where SD(X_i_) is the standard deviation of a patch. As patches synchronize, the value of φ_T_ grows from zero to one. The counterpart of inter-patch synchrony is persistence where, instead of temporal changes being similar from patch *i* to *j*, spatial differences are similar from time *k* to time *l*. We therefore estimated persistence with the spatial counterpart of φ_T_, which we call φ_S_. φ_S_ was calculated by replacing Var(Y) and SD(X_i_) in Eq. 1 with their spatial equivalents; the variance of the temporally-aggregated series (i.e., Var(Z)) and spatial standard deviation at time *k* (SD(X_k_)). Analogous to φ_T_, φ_S_ values increase from zero to one as differences among patches persist more through time.

We used General Linear Models (GLM) and multiple regression in Statistica v. 8.0 (StatSoft Inc., 2007) to predict regional temporal CV from mean spatial CV, φ_T_, and φ_S_ (i.e., ∼Fig. 1B). Temporal and spatial CV values were log transformed for analysis because, when plotted, they tended to form fan-shaped data clouds that were best described by power functions. We tested residuals of all analyses for normal distribution using Kolmogorov-Smirnov tests. Surfaces (Fig. 5) were fitted by distance-weighted least squares.

### Microcosm Connectivity Experiment

We assembled replicate arrays of three ×700 mL aquatic microcosms. Each array contained community types that were relatively stable under laboratory conditions: (i) impoverished, containing ubiquitous microbes initially surviving in distilled water, (ii) phytoplankton and microbes, and (iii) 10 invertebrate species (cladocerans, ostracods) and phytoplankton and microbes. We arranged microcosms such that each initially contained a distinct community type, with all three types represented in an array.

Spatial exchange was manipulated by connecting component microcosms with clear Tygon® tubes. Treatments were: No connection among microcosms and bi-directional connection among all three microcosms. Connector tube diameters were increased by ∼70% at week 10 of the 20-week experiment. Seven ecosystem-level variables were measured weekly in each microcosm of the array for 20 weeks using light-dark bottle methods, chlorophyll extractions and environmental sensor probes (Table S1). Microcosm NPP data were rescaled, bringing the lowest value to zero to correct spatial and temporal CV’s for negative values.

### Natural Rock Pool Ecosystem

We collected data over thirteen annual surveys (1989–2002) in a Jamaican rock-pool system of 78 invertebrate species, dispersing among 49 rock pools. The system lies near Discovery Bay Marine Laboratory, University of the West Indies, on the northern coast of Jamaica (18°28′ N, 77°25′ W). Pools create a mosaic 25 m in radius on a fossil reef no further than 10 m from the ocean and have volumes ranging from 0.5 to 78.4 L. Pools are, on average, within 1 m of the nearest neighbor and never more than 5 m away. Ocean tides occasionally flood a few of the most seaward pools. But most are refilled only by precipitation or, on some occasions, ocean spray. We treated the 49 pools as a single system linked by material fluxes and organism dispersal.

**Figure 6:**
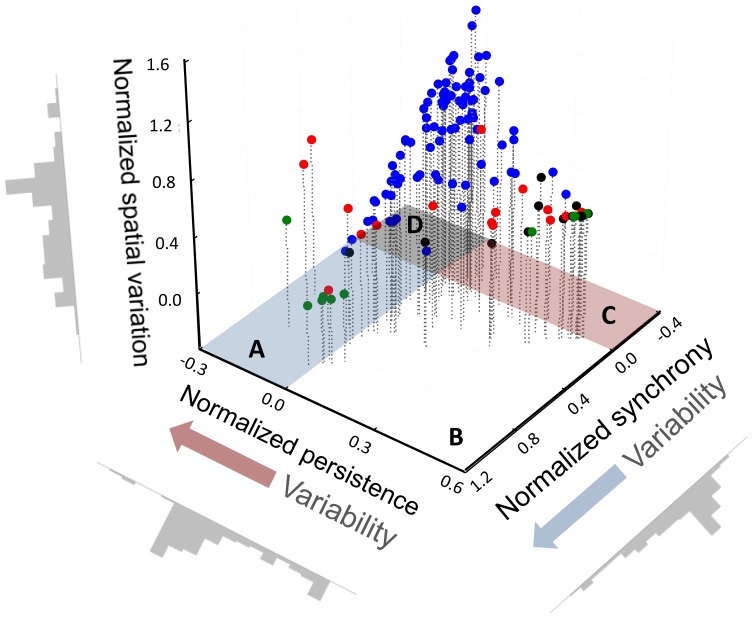
Patterns of spatiotemporal variation underlying the temporal variability of ecosystem variables. The interplay of the three components of temporal variance - spatial variation, synchrony and persistence - was captured by plotting the normalized values of each term in Fig. 1A against each other. Values of each term were standardized to the sum of all three terms such that the resulting proportions summed to one. Variables were assigned to *a priori* groupings based on their likely genesis and mode of regulation, where blue points = species populations, green = atmospheric, red = non-population biotic, black = watershed. n = 136, and includes an additional 36 rare rock pool species that were excluded from earlier analyses due to sparseness of data. Points scatter across theorized modes of dynamics described in Table 1: A = destabilized by synchrony, B = stabilized by persistence, destabilized by synchrony, C = stabilized by persistence, D = stabilized by compensatory dynamics, Intersection of A-D = stabilized by asynchrony. Gray histograms show frequency distributions for each component of temporal variance.

The 70+ invertebrate species in rock pools disperse predominantly by propagules transported via wind, ocean spray, animal vectors and, very occasionally, by overflow from neighboring pools after heavy rainfall [36]. Invertebrate species include: Ostracods (20 species), copepods (five species), cladocerans (five species), worms (15 species), aquatic insects (18 species) and other crustaceans (six species). Most species occurred rarely, some only once (for more details, see [37]). We therefore confined all analyses, except for contributions of variance components (Fig. 6) to 26 common species and temperature, pH, salinity, dissolved oxygen, oxygen saturation, and chlorophyll-a.

Invertebrate densities were estimated for each pool as the number of animals in a 0.5 L sample of water, which was withdrawn after stirring the pool to dislodge organisms from rock walls and to homogenize contents. Each sample was filtered through 63 µm mesh to isolate invertebrates, which were immediately preserved in 50% ethanol. Community samples were sorted, identified to highest possible taxonomic resolution and counted by microscope.

Environmental variables like salinity and pH (Table S2) were measured in each pool using multiprobe sondes (DataSonde, Yellow Springs Instruments, Yellow Springs, Ohio, USA or Hydrolab, Austin, Texas, USA) during biotic surveys for 6–11 of the survey years, depending on the variable.

Small rock pools occasionally dried up, preventing community sampling. These events were recorded as blank data entries, and were <10% of total observations. For our main analyses (Figs. 4–5), we replaced blank entries with zeroes, assuming that a desiccated pool harbored no living, adult invertebrates. To check if this assumption introduced bias, analyses were repeated using two alternative procedures; (i) leaving blank cells unchanged or (ii) interpolating by replacing blanks with the pool mean. All procedures produced similarly significant results indicating no major effect of our assumption. For abiotic variables, blank entries had no logical association with zero (e.g., desiccation does not suggest a 0°C temperature), so these cells were left blank.

### North Temperate Lakes Long-Term Ecosystem Research Program

We used data from seven Wisconsin lakes (Allequash, Big Muskellunge, Crystal, Sparkling, Trout, Crystal Bog and Trout Bog), collected by the North Temperate Lakes Long-Term Ecological Research program. Lake data were obtained from a public database hosted by the North Temperate Lakes LTER, NSF, Center for Limnology, University of Wisconsin-Madison, available at http://lter.limnology.wisc.edu. We included up to 30 years of data from 60 biotic and abiotic variables across five datasets (Table S2). The following datasets, collected and maintained by LTER associates, were used:

- Chemical limnology of primary study lakes: Major ions
- Chemical limnology of primary study lakes: Nutrients, pH and carbon
- Physical limnology of primary study lakes
- Pelagic macroinvertebrate abundance
- Fish abundance.

Collection methods corresponding to datasets can be found as metadata on the online database (http://lter.limnology.wisc.edu). For fish data, only fyke net catches were used and were standardized by effort (i.e., catch per unit effort) to facilitate comparison. Values for a given lake were annual, obtained by averaging organism densities or physicochemical values across depths, across sampling dates and across stations. Density data were used to equalize the contribution from each lake because Trout Lake is up to 3200 times larger than other lakes, and therefore dominates the landscape spatiotemporal pattern. Results therefore emphasize patterns owing to ecological differences among lakes, rather than to lake size.

Data are available from the LTER database or upon request from the authors.

## Results

Three quantities jointly accounted for 87–100% of a variable’s regional temporal CV (Table S3) - mean spatial CV, inter-patch synchrony φ_T,_ and persistence φ_S_. This result verifies that spatial and temporal CV’s are related and substitutable to the degree that synchrony or persistence does not interfere. Perfect substitution occurs when values are uncorrelated between patches *i* and *j* and between times *k* and *l.* In this case, regional temporal CV is roughly 1/n_i_^1/2^ times the mean spatial CV (Fig. 1B; Eq. S31). A null, stochastic model (see File S1) showed that such a variable (data point) lies within an “independent dynamics region” on a plot of spatial and temporal CV (Fig. 3A). It falls on a line of slope ∼1/n_i_^1/2^ or ∼1 on a log-scale (Fig. 3C). Inter-patch synchrony (e.g., from climatic forcing) increases regional temporal variability, shifting a variable to an upper “synchrony region” of the plot (Fig. 3A). But the data point shifts to a lower, “persistence region” (Fig. 3A) when spatial gradients are retained over time (e.g., from patch-specific factors).

A range of plot patterns can emerge depending on the variables sampled and their spatiotemporal dynamics. First, weak or no linear association exists when variables’ spatial and temporal CV’s span only a narrow range and stochastic differences in synchrony and persistence create scatter (Fig. 3B). Second, strong linear association emerges if all landscape variables have stochastic behavior (black circles, Fig. 3C), similar degrees of inter-patch synchrony (blue circles, Fig. 3C) or similar degrees of persistence (red circles, Fig. 3C). The latter two cases occur because all points are equally displaced up or down from the independent dynamics region. Finally, regression slopes deviate from the expected slope of 1 when some variables display synchrony and others persistence to produce skew (Fig. 3D).

Significant linear regressions existed between spatial CV and regional temporal CV for real ecosystem variables (Fig. 4). In all three data sets, the most stable variables (e.g., hydrological and environmental variables) had low spatial variability, while unstable variables (e.g., species populations) were spatially patchy in the landscape (Fig. 4A–C). Some statistical dependence exists in these plots because mean spatial CV and regional temporal CV are calculated from the same data matrix. However, results were almost perfectly conserved when a single sampling event (*k*) served to estimate spatial CV and predict subsequent (*k+1…n*) temporal CV (Fig. 4D–F). Results are also unlikely to have arisen from biased estimators, since trends were confirmed using several alternative indices (Table S3).

Not all variables lay close to the independent dynamics region. This was reflected in regression slopes and intercepts (solid lines, Fig. 4) which departed from the independent dynamics case (dashed lines, Fig. 4). For instance, regression slopes for microcosms and rock pools were reduced because physicochemical variables (blue circles) at one end of the data cloud exhibited more inter-patch synchrony (Fig. 4A,B,D,E). Meanwhile, lake physicochemical variables showed considerable persistence, lowering temporal variability and steepening the slope (Fig. 4C,F). Though slopes and intercepts deviated from the independent dynamics region, r^2^ values of 0.70–0.93 suggest that the rank order of variable’s CV’s was preserved. We do note some outliers, however, such as pH, Mg and Ca in the persistence region of Fig. 4C, F. Also, some variable types (e.g., species populations; red circles) had greater scatter. Overall, however, synchrony and persistence interfered relatively little with the scaling of spatial and temporal CV. Multiple-regression beta coefficients, for instance, revealed that temporal CV increased 2.9–6.2 times more with a variable’s spatial CV than with its synchrony (Fig. 5).

Variables differed in the relative importance of spatial variability, synchrony or persistence to their landscape dynamics. We generated a fingerprint of these dynamics by normalizing the right hand terms of the variance equation (Fig. 1A) for each variable and then plotting them (Fig. 6). A division existed between biotic and physicochemical dynamics, and variables spread across several regions of the plot (quadrants A-D) corresponding to different spatiotemporal behaviors leading to temporal variability. Table 1 synthesizes results by describing these modes of behavior and how they may arise in nature.

**Table 1:** Theorized modes of dynamics in landscape variables, their effect on regional temporal variation, and ecological examples.

Graph region	Synchrony/persistence	Dominant effect ontemporal variation	Ecological scenario
A	High/Low	Destabilized by synchrony	Local factors less important, landscape-scale factors synchronize dynamics. E.g., synchrony of isolated mammal populations via weather [63]
B	High/High	Stabilized by persistence and destabilized by synchrony	Local factors establish permanent spatial gradients, landscape-scale factors synchronize dynamics. E.g., synchrony of source-sink fish populations via dispersal [64]
C	Low/High	Stabilized by persistence	Local factors establish permanent spatial gradients, dynamics differ among sites. E.g., stable spatial distributions of organisms across habitats [65]
D	Low/Low	Stabilized by compensatory dynamics	Dynamics negatively correlated from time to time, site to site. E.g., spatiotemporal refugia of competing soil nematodes [60]
Intersection ofA,B,C,D	Zero/Zero	Stabilized by asynchrony	Dynamics appear stochastic, independent from time to time, site to site. E.g., settlement of broadcast oceanic larvae [66]

Modes reflect different mixtures of inter-patch synchrony and persistence, and correspond to regions of Fig. 6, where variables from three natural ecosystems are plotted by their spatiotemporal patterns.

Species populations (blue points) clumped together, their dynamics dominated by spatial variability with little synchrony or persistence (Fig. 6; intersection of quadrants A-D). Meanwhile, most atmospherically-driven processes (green points e.g., temperature, dissolved oxygen) were set apart by little persistence but were destabilized by synchrony (quadrant A). In contrast, many watershed-associated variables (black points e.g., ion concentrations) were characterized by persistence but little synchrony (quadrant B), a combination leading to lower temporal variability at the regional scale.

## Discussion

### Spatial Signatures of Temporal Variability

Variables from three aquatic ecosystems showed a striking and tight correspondence between their regional temporal CV and mean spatial CV. This trend may be considered predictive because it held even when the spatial CV was known from only one time point. Moreover, trends emerged in ecosystems ranging from large to small, and from tropical to temperate, suggesting a potentially general and widespread phenomenon. Applying a space-time correspondence follows more than a century of studies involving substitution [8]–[10], [38], but our formulation extends usefulness in two ways; (i) it is quantitative in the form of equations in Fig. 1 rather than qualitative (e.g., chronosequence studies; see [33] for critical review) and (ii) the logic applies equally when substituting CV’s of a single variable or when plotting many ecosystem variables for a multivariate view of landscape variation.

Tight linear dependence between spatial and temporal CV’s likely owes to two reasons: First, when dynamics are stochastic or independent, variation in space roughly matches that in time as in ergodicity. Thus small fluctuations in time render equally small fluctuations across space. Second is the empirical observation that the factors which theoretically interfere with this correspondence - synchrony and persistence - do so little, at least when using the CV. While variables can lie anywhere on the plot (shifted up the y-axis by synchrony, down by persistence; Fig. 3C), they adhered more to the “independent dynamics” region than being shifted (Fig. 5). This makes sense in that a variable (e.g., a population) with low temporal CV in each patch will still be relatively stable regionally even when patches partially synchronize. This, in turn, registers as a low spatial CV because of how small temporal fluctuations beget small spatial variation (Fig. 2). There are exciting hints that this type of correspondence also applies to other temporal properties, such as recovery time or deterministic chaos, that leave a telling trace of their temporal dynamics in space [13], [16].

Analytical solutions and simulations show that spatial CV has value as a signature of temporal variability under certain conditions. When values of a variable are relatively uncorrelated in time and space (e.g., Fig. 4A), the temporal CV can be recovered with an analytical approximation (Fig. 1B; Eq. S31). Accuracy wanes when ecological forces synchronize patches and shift the variable into the synchrony region of the plot (Fig. 3) where spatial CV underestimates temporal CV. Here, synchrony simultaneously boosts temporal variability and lowers spatial variability by aligning the peaks and troughs of fluctuations. Accuracy is also lower when ecological forces cause spatial gradients to persist through time and shift a variable into the persistence region of the plot (Fig. 3). Here, spatial variability exists, but it is created by patches that are stable over time.

Dynamics must be reasonably assumed to be stochastic to use spatial CV as a quantitative proxy for temporal CV. This assumption will often not hold in nature (e.g., when climate swings induce synchrony). And whether it does hold will likely depend on the types of variables chosen (fast or slow, broad-scale or fine-scale) as well as the spatial and temporal sampling scales (Table 2). Even when the assumption does not hold, however, regressions suggest that the rank order of temporal CV’s (e.g., highest to lowest) might still be recovered from spatial CV’s for qualitative substitution. This should be possible when: (i) All variables are thought to be synchronized or persistent to the same degree (Fig. 3C), (ii) they smoothly intergrade from synchrony to persistence (Figs. 3D; 4C,F), and/or (iii) the distorting effect of synchrony and persistence can be estimated and corrected for (Table 2). Thus, the link between spatial and temporal variation may be valuable for understanding when CV’s are interchangeable, and how to interpret them when they are not.

**Table 2:** Conditions under which the spatial variability of an ecological process is a precise or accurate substitute for its regional temporal variability.

Condition	Description	Substitution	Requirements	Ecological scenario
Independent dynamics	Values must be reasonably assumedindependent from place to place,time to time	Spatial and temporal CV values roughly interchangeable (see Fig. 1B)	Scale of process ≤ scale of measurement in both space and time	Isolated communities with fast dynamics; intercontinental, long-term comparisons
Constant levels ofsynchrony orpersistence acrossvariables	Spatiotemporal behavior (e.g.,synchrony) that is shared by allvariables shifts all points equallyon plot (Fig. 3C). Values shiftedby a constant	Order (rank) of temporalCV’s is conserved amongvariables	Approximately equivalent spatiotemporal behavior among variables	Variables similarly shaped by spatial constraints (e.g., microclimates) or by landscape-scale temporal shocks (e.g., weather)
Mixed synchrony and persistence of known magnitude	Variables differ in their synchronyand persistence (Fig. 3D), butdegree of divergence is knownand corrected for	Temporal CV value ororder (rank) can beback-calculated	Estimates of synchronyand/or persistence; Fairlystationary dynamics	Variables responding differentially to spatiotemporal variation in the landscape with synchrony or persistence

Some variable types may be better suited to substitution than others. Interestingly, species populations (Fig. 4; red circles) illustrated a tradeoff between precision and accuracy. Precision to distinguish species with high versus low temporal CV’s using spatial CV’s was weak. This was for the sampling reasons that species had a limited range of variability (i.e., range of CV’s<scatter) or possibly greater measurement error; or for the ecological reason that they differed in synchrony or persistence which created scatter. Such differences can occur when species respond even slightly differently to the spatial over the temporal environment [39]. But on the other hand, accuracy was probably higher for species because these variables lay closer to the independent dynamics line than physicochemical variables.

Results captured strong associations between spatial and temporal CV when data were separated by one time point (Fig. 4C–F) or were overlapping (Fig. 4A–C). Future work must address how far into the future a spatial CV can be a proxy for temporal CV. Additional error will certainly accrue for substitution if unexpected events alter a variable’s temporal variability in ways not indicated by its initial spatial variability. The exact impact of this non-stationarity, however, is a matter of scale and of research question asked. For instance, high resolution prediction of single-species dynamics following a stochastic disturbance (e.g., forest fire) may be untenable if the disturbance drastically alters variability, synchrony or spatial persistence. But spatial CV should be a rough proxy – either quantitative or qualitative – for regional temporal CV under the ecological and sampling conditions in Table 2.

### Diagnostic Signatures of Complex Dynamics

The terms of Fig. 1 also allowed a window into diagnosing landscape dynamics. Ecosystems conceal ecological information in an eclectic range of spatiotemporal patterns [40], [41]. Populations of exploited species [42], biodiversity hotspots [43], harmful algal blooms [44], pest outbreaks [45] and wildfires [46] all display complex patterning in space and time. A range of analyses explore these patterns by examining underlying frequencies (e.g., spectral [47] and wavelet analysis [48]) and patterns of correlation (e.g., correlograms [49]), or by fitting predictive models (e.g., autoregressive [50]). But these approaches are not designed for linking spatiotemporal pattern to a variable’s temporal variation.

We used plots of spatial versus temporal CV’s as convenient and unique summaries of landscape dynamics (Fig. 4). Some variables occupied the synchrony region (above dashed line), others the persistence region (below dashed line). Such a mixture may be typical when a wide array of ecosystem processes is sampled. These mixtures left their mark on regression slopes. Slopes <1 in Fig. 4A–B show that abiotic variables in microcosms and rock pools were more stable, but also more prone to synchrony, than populations. Y-intercepts also changed to the degree that multiple variables displayed synchrony or persistence. These therefore offer multivariate indices of the degree of synchrony (β_0_> expected) or persistence (β_0_< expected) experienced by a landscape (Fig. 3C). Such plots may be fruitful ground for streamlined comparisons of landscapes containing diverse variables and dynamics, like pre-and post-disturbance ecosystems.

The components of temporal variance themselves – spatial variance, synchrony and persistence (Fig. 1A) – may also prove useful for describing patterns and mechanisms driving temporal variation. As we have seen, spatial variance can signal instability at the local, patch scale (e.g., from demographic [51], community [52], natural enemies [53], spatial [26] or local environmental causes; Table S4). Synchrony, in turn, indicates landscape-scale causes of variation like dispersal [53], [54] or weather [55], [56]. Finally, persistence points to the existence of long-term differences in mean value or state among patches, such as regulation by local communities or physical conditions. Combined, these components of variation gave an alternative view of dynamics.

Fine distinctions emerged when all elements of Fig. 1A were normalized to create a fingerprint or signature of dynamics. The grouping of variables controlled by different parts of the biosphere, like the atmosphere (e.g., temperature, dissolved oxygen) and the watershed (e.g., pH, ion concentrations), suggests that unique signatures may exist for types of ecological processes. Potential may thus exist for predicting the likely dynamics of a variable based on its type (e.g., atmospheric). Meanwhile, the breadth of spatiotemporal behaviors seen suggests a range of spatial variation, synchrony and persistence combinations leading to temporal variation in nature. Our framework may be useful for cataloguing these types of spatiotemporal dynamics in ecosystems (e.g., Table 1), and for making broad-stroke inferences about spatiotemporal mechanisms (e.g., population rescue effects [26], [57], predator-prey cycles [58], species coexistence [59], [60], and invasive species spread [61]).

## Conclusions

Unexplained variation is common in nature, both across heterogeneous landscapes and over timespans of interest. By illustrating the link between spatial and temporal variation, we bring more clarity to the problems of *prediction* and *diagnosis* from spatial or spatiotemporal patterns. More work is needed to test the limits of prediction across scales, variables and ecosystem types. Yet, indications here suggest usefulness in; substituting spatial for temporal variability (either quantitatively or qualitatively), judging when substitution will not work, and interpreting the manifold changes of multivariate landscapes. Such efforts are hoped to add momentum towards the Rosetta Stone of landscape and ecosystem ecology, in which process and mechanism may be deeply and easily discerned from landscape pattern [62].

## Supporting Information

File S1 (DOCX)

Table S1 **Community and ecosystem variables measured over the 20-week microcosm connectivity experiment.**
(DOCX)

Table S2 **Variables from lake, rock pool and microcosm data sets used in analyses.** An additional 36 rock pool species (not shown), known from fewer occurrences, were included for calculating spatiotemporal signatures (Fig. 6).
(DOCX)

Table S3 **Comparison of CV-based results with alternative indices of variability.** General Linear Models were fit between indices of aggregate temporal variability and three spatiotemporal descriptors: Spatial variability, inter-patch synchrony and persistence (see Fig. 1B). Indices of variability included CV and four others. Asterisks denote statistical significance and p-value. *p<0.05, **p<0.01, ***p<0.001. R^2^ values in parentheses.
(DOCX)

Table S4 **Mechanisms that may dampen regional variability and reduce spatial variability by stabilizing local patches.**
(DOCX)
