# Supplementary material for: Spatial Variation as a Tool for Inferring Temporal Variation and Diagnosing Types of Mechanisms in Ecosystems
Source: PLoS One. 2014 Feb 20;9(2):e89245. doi: 10.1371/journal.pone.0089245 (PMC3930753; doi:10.1371/journal.pone.0089245)
Supplement: File S1 — (DOCX) [file pone.0089245.s001.docx]

**Supporting Information for:** *Spatial variation as a tool for inferring temporal variation and diagnosing types of mechanisms in ecosystems*

Authors: Matthew P. Hammond, Jurek Kolasa

1. Analytical expression of temporal variance in spatial terms

*X_ik_* denotes the value of an ecosystem variable at patch *i* and time *k.* Each patch *i* has a temporal variance var(X_i_) from a time series of *n_k_* time points. Each time point *k* has an associated spatial variance var(X_k_) from a spatial series of *n_i_* patches. Regional variance is the variance of the spatially-aggregated time series (i.e., Var(Y) where$Y_{k}=\sum_{i=1}^{n}X_{ik}$), and reflects variation at the aggregate (i.e., landscape) scale. Here we analytically express this variance in terms of spatial variance var(X_k_).

Temporal variation can also be expressed as a portion of the data matrix’s total variation (SS_Tot_), using sums of squares (SS) in ANOVA definitional formulas [1]:

| $\text{SS}_{\text{Tot}}=\text{ SS}_{\text{K}}+\text{SS}_{\text{within K}}$ |  | Eq. S1 |
| --- | --- | --- |

where SS_K_ is the variation *between* time points *k* and *l*, and SS_within K_ is the variation *within* time point *k* (i.e., of values across space).

Note that total variation of the data matrix can equivalently be partitioned with space as a factor instead of time. This casts SS_Tot_ in terms of variation *between* patches *i* and *j* (SS_I_) and variation *within* patch *i* (SS_within I_; i.e., variation of values over time):

| $\text{SS}_{\text{Tot}}=\text{ SS}_{\text{I}}+\text{SS}_{\text{within I}}$ |  | Eq. S2 |
| --- | --- | --- |

Substituting SS_Tot_ in Eq. S2 for the same in Eq. S1, we equate components of spatial and temporal variation:

| $\text{SS}_{\text{K}}+\text{SS}_{\text{within K}}=\text{ SS}_{\text{I}}+\text{SS}_{\text{within I}}$ |  | Eq. S3 |
| --- | --- | --- |

SS terms can also be expressed as spatial and temporal variances. For instance, the variance analogue of variation between time points (SS_K_) is regional temporal variance Var(Y). Meanwhile, the equivalent of variation between patches (SS_I_) is an aggregate variance Var(Z), which is the variance of the temporally-aggregated series (i.e., $\text{Z}_{\text{i}}=\sum_{k=1}^{n}\text{X}_{ik}$). We converted all SS terms into temporal and spatial variances according the identities in Eqs. S4-S7. These identities were obtained by first converting SS to mean sums of squares, and then adjusting for computational differences arising from using sums (as for aggregate variances, Var(Y) and Var(Z)) as opposed to using means (as for SS).

| $\text{SS}_{\text{K}}=\frac{\text{n}_{\text{k}}-1}{\text{n}_{\text{i}}}\text{ Var}\left(\text{Y}\right)$ |  | Eq. S4 |
| --- | --- | --- |
| $\text{SS}_{\text{I}}=\text{ }\frac{\text{n}_{\text{i}}-1}{\text{n}_{\text{k}}}\text{Var}\left(\text{Z}\right)$ |  | Eq. S5 |
| $\text{SS}_{\text{within K}}=\text{ }\left(\text{n}_{\text{i}}-1\right)\sum_{\text{k}=1}^{\text{n}} \text{var}\left(\text{X}_{\text{k}}\right)$ |  | Eq. S6 |
| $\text{SS}_{\text{within I}}=\text{ }\left(\text{n}_{\text{k}}-1\right)\sum_{\text{i}=1}^{\text{n}} \text{var}\left(\text{X}_{\text{i}}\right)$ |  | Eq. S7 |

Substituting the identities (Eq. S4-S7) into Eq. S3 and rearranging, we obtain:

| $\frac{\text{n}_{\text{k}}-1}{\text{n}_{\text{i}}}\text{ Var}\left(\text{Y}\right)=\text{ }\left(\text{n}_{\text{k}}-1\right)\sum_{\text{i}=1}^{\text{n}} \text{var}\left(\text{X}_{\text{i}}\right)-(\text{n}_{\text{i}}-1)\sum_{\text{k}=1}^{\text{n}} \text{var}\left(\text{X}_{\text{k}}\right)+\frac{\text{n}_{\text{i}}-1}{\text{n}_{\text{k}}}\text{Var}\left(\text{Z}\right)$ |  | Eq. S8 |
| --- | --- | --- |

We may simplify by decomposing the temporal and spatial aggregate variances, Var(Y) and Var(Z), into yet more components of variation. It has long been recognized that any aggregate variance is a sum of the variances of its components and their covariances [2], as follows for the case of regional temporal variance, Var(Y):

| $\text{Var}\left(\text{Y}\right)=\text{ }\sum_{\text{i}=1}^{\text{n}} \text{var}\left(\text{X}_{\text{i}}\right)+2\sum_{\text{i}=1}^{\text{n}} \sum_{\text{j}=1}^{\text{i}-1} \text{cov}\left(\text{X}_{\text{i}},\text{X}_{\text{j}}\right)$ |  | Eq. S9 |
| --- | --- | --- |

where var(X_i_) is the temporal variance of patch *i* and cov(X_i_, X_j_) is the covariance of patch *i* with patch *j*.

An analogous decomposition applies to aggregate spatial variance Var(Z):

| $\text{Var}\left(\text{Z}\right)=\text{ }\sum_{\text{k}=1}^{\text{n}} \text{var}\left(\text{X}_{\text{k}}\right)+2\sum_{\text{k}=1}^{\text{n}} \sum_{\text{l}=1}^{\text{k}-1} \text{cov}\left(\text{X}_{\text{k}},\text{X}_{\text{l}}\right)$ |  | Eq. S10 |
| --- | --- | --- |

where var(X_k_) is the spatial variance observed at time *k*, and cov(X_k_, X_l_) is the covariance between spatial series at times *k* and *l*.

Substituting the definition of aggregate spatial variance from Eq. S10 into Eq. S8, we express Var(Y) in part as spatial variances and covariances. Then, we rearrange Eq. S9 to isolate ∑var(X_i_) - the summed temporal variances of patches - and substitute this into the result of the preceding step to yield:

| $\frac{\text{n}_{\text{k}}-1}{\text{n}_{\text{i}}}\text{ Var}\left(\text{Y}\right)=\text{ }(\text{n}_{\text{k}}-1)\text{Var}\left(\text{Y}\right)-(\text{n}_{\text{i}}-1)\sum^{ } \text{var}\left(\text{X}_{\text{k}}\right)+\left(\frac{\text{n}_{\text{i}}-1}{\text{n}_{\text{k}}}\right)\sum^{ } \text{var}\left(\text{X}_{\text{k}}\right)-\left(2\text{n}_{\text{k}}-1\right)\sum^{ } \text{cov}\left(\text{X}_{\text{i}},\text{X}_{\text{j}}\right)+\left(\frac{2\text{n}_{\text{i}}-1}{\text{n}_{\text{k}}}\right)\sum^{ } \text{cov}\left(\text{X}_{\text{k}},\text{X}_{\text{l}}\right)$ |  | Eq. S11 |
| --- | --- | --- |

Rearranging and simplifying, we define aggregate temporal variance exactly in terms of spatial variances, covariances among patches, and covariances among time points:

| $\text{Var}\left(\text{Y}\right)=\frac{\text{n}_{\text{i}}}{\text{n}_{\text{k}}}\sum_{\text{k}=1}^{\text{n}} \text{var}\left(\text{X}_{\text{k}}\right)+\frac{2\text{n}_{\text{i}}}{\text{n}_{\text{i}}-1}\sum_{\text{i}=1}^{\text{n}} \sum_{\text{j}=1}^{\text{i}-1} \text{cov}\left(\text{X}_{\text{i}},\text{X}_{\text{j}}\right)-\frac{2\text{n}_{\text{i}}}{\text{n}_{\text{k}}^{2}-\text{n}_{\text{k}}}\sum_{\text{k}=1}^{\text{n}} \sum_{\text{l}=1}^{\text{k}-1} \text{cov}\left(\text{X}_{\text{k}},\text{X}_{\text{l}}\right)$ |  | Eq. S12 |
| --- | --- | --- |

As in Eq. S9, aggregate variance increases when patches covary in time (∑cov(X*_i_,*X*_j_*)). Here, however, two spatial terms replace the term corresponding to the temporal variances of patches (∑var(X_i_)). These two terms depict the spatial signature left behind when local patches vary (∑var(X_k_)), and a stabilizing effect when spatial variation persists over time (∑cov(X*_k_,*X*_l_*)).

2. Analytical expression of regional temporal CV in terms of spatial CV

Eq. S12 may be re-expressed in dimensionless terms from which the effect of the mean is removed. Below we derive an exact expression that incorporates commonly-used Coefficients of Variation (CV) as well as φ indices of patch synchrony or persistence of spatial variation.

We first divide Eq. S12 by the square of the mean of the regional temporal series$\overline{\text{X}_{\text{Y}}}$to get:

| $\frac{\text{Var}\left(\text{Y}\right)}{\left(\overline{\text{X}_{\text{Y}}}\right)^{2}}=\text{ }\frac{\text{SD}_{\text{Y}}}{\overline{\text{X}_{\text{Y}}}}\cdot\frac{\text{SD}_{\text{Y}}}{\overline{\text{X}_{\text{Y}}}}=\text{CV}^{2}=\frac{\text{n}_{\text{i}}}{\text{n}_{\text{k}}}\frac{\sum^{ } \text{var}\left(\text{X}_{\text{k}}\right)}{\left(\overline{\text{X}_{\text{Y}}}\right)^{2}}+\frac{2\text{n}_{\text{i}}}{\text{n}_{\text{i}}-1}\frac{\sum^{ } \text{cov}\left(\text{X}_{\text{i}},\text{X}_{\text{j}}\right)}{\left(\overline{\text{X}_{\text{Y}}}\right)^{2}}-\frac{2\text{n}_{\text{i}}}{\text{n}_{\text{k}}^{2}-\text{n}_{\text{k}}}\frac{\sum^{ } \text{cov}\left(\text{X}_{\text{k}},\text{X}_{\text{l}}\right)}{\left(\overline{\text{X}_{\text{Y}}}\right)^{2}}$ |  | Eq. S13 |
| --- | --- | --- |

where *n_i_* and *n_k_* are numbers of patches and time points, respectively, and SD_Y_ is the regional standard deviation. We then substitute variance and covariance terms for dimensionless coefficients.

A. Converting spatial variance term (∑var(X_k_)) into spatial CV’s

From [3] it is known that maximum aggregate variance occurs when covariance among components is highest and is equivalent to the square of their summed standard deviations. The maximum variance of a temporally-aggregated series is thus:

| $\text{Var}\left(\text{Z}\right)_{\text{max}}=\left(\sum_{\text{k}=1}^{\text{n}} \text{SD}\left(\text{X}_{\text{k}}\right)\right)^{2}$ |  | Eq. S14 |
| --- | --- | --- |

W the spatial standard deviation at time where SD(X_k_) is the spatial standard deviation at time *k*. Dividing by ∑SD(X_k_) and expressing Var(Z)_max_ as summed spatial variances and covariances (Eq. S10), we get:

| $\text{ }\sum^{ } \text{SD}\left(\text{X}_{\text{k}}\right)=\frac{\text{Var}\left(\text{Z}\right)_{\text{max}}}{\sum^{ } \text{SD}\left(\text{X}_{\text{k}}\right)}=\frac{\sum^{ } \text{var}\left(\text{X}_{\text{k}}\right)+2\sum^{ } \text{cov}\left(\text{X}_{\text{k}},\text{X}_{\text{l}}\right)_{\text{max}}}{\sum^{ } \text{SD}\left(\text{X}_{\text{k}}\right)}\text{ }$ |  | Eq. S15 |
| --- | --- | --- |

Rearranging for ∑var(X_k_), we find:

| $\sum^{ } \text{var}\left(\text{X}_{\text{k}}\right)=\left(\sum^{ } \text{SD}\left(\text{X}_{\text{k}}\right)\right)^{2}-2\sum^{ } \text{cov}\left(\text{X}_{\text{k}},\text{X}_{\text{l}}\right)_{\text{max}}$ |  | Eq. S16 |
| --- | --- | --- |

Plugging this into the ∑var(X_k_) term of Eq. S13 yields:

| $\frac{\text{n}_{\text{i}}}{\text{n}_{\text{k}}}\cdot\frac{\sum^{ } \text{var}\left(\text{X}_{\text{k}}\right)}{\left(\overline{\text{X}_{\text{Y}}}\right)^{2}}=\frac{\text{n}_{\text{i}}}{\text{n}_{\text{k}}}\left(\frac{\sum^{ } \text{SD}\left(\text{X}_{\text{k}}\right)}{\overline{\text{X}_{\text{Y}}}}\cdot\frac{\sum^{ } \text{SD}\left(\text{X}_{\text{k}}\right)}{\overline{\text{X}_{\text{Y}}}}-\frac{2\sum^{ } \text{cov}\left(\text{X}_{\text{k}},\text{X}_{\text{l}}\right)_{\text{max}}}{\left(\overline{\text{X}_{\text{Y}}}\right)^{2}}\right)$ |  | Eq. S17 |
| --- | --- | --- |

Re-writing the temporal mean$\overline{\text{X}_{\text{Y}}}$as the sum of spatial means at time *k*,$\sum^{ } \overline{\text{X}_{\text{k}}}$, we get:

| $\frac{\text{n}_{\text{i}}}{\text{n}_{\text{k}}}\cdot\frac{\sum^{ } \text{var}\left(\text{X}_{\text{k}}\right)}{\left(\overline{\text{X}_{\text{Y}}}\right)^{2}}=\frac{\text{n}_{\text{i}}}{\text{n}_{\text{k}}}\left(\frac{\sum^{ } \text{SD}\left(\text{X}_{\text{k}}\right)}{\frac{\text{n}_{\text{i}}}{\text{n}_{\text{k}}}\sum^{ } \overline{\text{X}_{\text{k}}}}\cdot\frac{\sum^{ } \text{SD}\left(\text{X}_{\text{k}}\right)}{\frac{\text{n}_{\text{i}}}{\text{n}_{\text{k}}}\sum^{ } \overline{\text{X}_{\text{k}}}}-\frac{2\sum^{ } \text{cov}\left(\text{X}_{\text{k}},\text{X}_{\text{l}}\right)_{\text{max}}}{\left(\overline{\text{X}_{\text{Y}}}\right)^{2}}\right)$ |  | Eq. S18 |
| --- | --- | --- |

The first term within brackets divides summed spatial standard deviations by summed spatial means. [4] point out that this quantity is equivalent to a weighted-mean CV,$\widetilde{\text{CV}}_{k}$, where each spatial CV at time *k* is weighted by its relative mean:

| $\widetilde{\text{CV}}_{\text{k}}=\sum_{k=1}^{n} \frac{\bar{\text{X}}_{\text{k}}}{\sum_{\text{l}} \bar{\text{X}}_{\text{l}}}\text{CV}_{\text{k}}$ |  | Eq. S19 |
| --- | --- | --- |

Therefore:

| $\frac{\text{n}_{\text{i}}}{\text{n}_{\text{k}}}\cdot\frac{\sum^{ } \text{var}\left(\text{X}_{\text{k}}\right)}{\left(\overline{\text{X}_{\text{Y}}}\right)^{2}}=\frac{\text{n}_{\text{k}}}{\text{n}_{\text{i}}}\widetilde{\text{CV}}_{\text{k}}^{2}-\frac{\text{n}_{\text{i}}}{\text{n}_{\text{k}}}\cdot\frac{2\sum^{ } \text{cov}\left(\text{X}_{\text{k}},\text{X}_{\text{l}}\right)_{\text{max}}}{\left(\overline{\text{X}_{\text{Y}}}\right)^{2}}$ |  | Eq. S20 |
| --- | --- | --- |

B. Converting inter-patch synchrony term (∑cov(X_i_, X_j_)) into φ_T_ index

The φ_T_ index is a dimensionless statistic of synchrony between patches *i* and *j*. It works by comparing observed aggregate-level variance to the theoretical maximum when patches are perfectly synchronized and is defined as:

| $\text{φ}_{\text{T}}=\frac{\text{Var}\left(\text{Y}\right)}{\left(\sum^{ } \text{SD}\left(\text{X}_{\text{i}}\right)\right)^{2}}=\frac{\text{Var}\left(\text{Y}\right)}{\text{Var}\left(\text{Y}\right)_{\text{max}}}=\frac{\sum^{ } \text{var}\left(\text{X}_{\text{i}}\right)+2\sum^{ } \text{cov}\left(\text{X}_{\text{i}},\text{X}_{\text{j}}\right)}{\sum^{ } \text{var}\left(\text{X}_{\text{i}}\right)+2\sum^{ } \text{cov}\left(\text{X}_{\text{i}},\text{X}_{\text{j}}\right)_{\text{max}}}$ |  | Eq. S21 |
| --- | --- | --- |

where SD(X_i_) is the temporal standard deviation of patch *i*. We can rearrange this expression to isolate ∑cov(X_i_, Xj) and simplify to:

| $\sum^{ } \text{cov}\left(\text{X}_{\text{i}},\text{X}_{\text{j}}\right)=\frac{1}{2}\left(\text{φ}_{\text{T}}\left(\sum^{ } \text{var}\left(\text{X}_{\text{i}}\right)+2\sum^{ } \text{cov}\left(\text{X}_{\text{i}},\text{X}_{\text{j}}\right)_{\text{max}}\right)-\sum^{ } \text{var}\left(\text{X}_{\text{i}}\right)\right)$ |  | Eq. S22 |
| --- | --- | --- |

Substituting this for the covariances in the second term of Equation S13 and simplifying, we link our variance-based formula to φ_T_:

| $\frac{2\text{n}_{\text{i}}}{\text{n}_{\text{i}}-1}\cdot\frac{\sum^{ } \text{cov}\left(\text{X}_{\text{i}},\text{X}_{\text{j}}\right)}{\left(\overline{\text{X}_{\text{Y}}}\right)^{2}}=\frac{\text{n}_{\text{i}}}{\text{n}_{\text{i}}-1}\cdot\frac{\text{φ}_{\text{T}}\text{Var}\left(\text{Y}\right)_{\text{max}}-\sum^{ } \text{var}\left(\text{X}_{\text{i}}\right)}{\left(\overline{\text{X}_{\text{Y}}}\right)^{2}}$ |  | Eq. S23 |
| --- | --- | --- |

C. Converting persistence term (∑cov(X_k_, X_l_)) into φ_S_ index

To eliminate the extra covariance term from Eq. S20, we combine it with the third term of Eq. S13 to yield:

| $\frac{2\text{n}_{\text{i}}}{\text{n}_{\text{k}}^{2}-\text{n}_{\text{k}}}\cdot\frac{\left(\left(\text{n}_{\text{k}}-1\right)\sum^{ } \text{cov}\left(\text{X}_{\text{k}},\text{X}_{\text{l}}\right)_{\text{max}}+\sum^{ } \text{cov}\left(\text{X}_{\text{k}},\text{X}_{\text{l}}\right)\right)}{\left(\overline{\text{X}_{\text{Y}}}\right)^{2}}$ |  | Eq. S24 |
| --- | --- | --- |

To introduce φ_S_ into the equation, we define it in terms of covariance of time point *k* with *l* (persistence). φ_S_ is the exact analogue of φ_T_, and so contrasts the variance of the temporally-aggregated series *Z* with the maximum possible aggregate variance when covariances of spatial series are highest:

| $\text{φ}_{\text{S}}=\frac{\text{Var}\left(\text{Z}\right)}{\left(\sum^{ } \text{SD}\left(\text{X}_{\text{k}}\right)\right)^{2}}=\frac{\text{Var}\left(\text{Z}\right)}{\text{Var}\left(\text{Z}\right)_{\text{max}}}=\frac{\sum^{ } \text{var}\left(\text{X}_{\text{k}}\right)+2\sum^{ } \text{cov}\left(\text{X}_{\text{k}},\text{X}_{\text{l}}\right)}{\sum^{ } \text{var}\left(\text{X}_{\text{k}}\right)+2\sum^{ } \text{cov}\left(\text{X}_{\text{k}},\text{X}_{\text{l}}\right)_{\text{max}}}$ |  | Eq. S25 |
| --- | --- | --- |

Rearranging for ∑cov(X_k_, X_l_), we find:

| $\sum^{ } \text{cov}\left(\text{X}_{\text{k}},\text{X}_{\text{l}}\right)=\frac{1}{2}\left(\text{φ}_{\text{S}}\left(\sum^{ } \text{var}\left(\text{X}_{\text{k}}\right)+2\sum^{ } \text{cov}\left(\text{X}_{\text{k}},\text{X}_{\text{l}}\right)_{\text{max}}\right)-\sum^{ } \text{var}\left(\text{X}_{\text{k}}\right)\right)$ |  | Eq. S26 |
| --- | --- | --- |

Substituting this for ∑cov(X_k_, X_l_) in Eq. S24 and simplifying, we express persistence from Eq. S13 in terms of φ_S_:

| $\frac{\text{n}_{\text{i}}}{\text{n}_{\text{k}}^{2}-\text{n}_{\text{k}}}\cdot\frac{\text{φ}_{\text{S}}\text{Var}\left(\text{Z}\right)_{\text{max}}-\sum^{ } \text{var}\left(\text{X}_{\text{k}}\right)+2\left(\left(\text{n}_{\text{k}}-1\right)\sum^{ } \text{cov}\left(\text{X}_{\text{k}},\text{X}_{\text{l}}\right)_{\text{max}}\right)}{\left(\overline{\text{X}_{\text{Y}}}\right)^{2}}$ |  | Eq. S27 |
| --- | --- | --- |

Writing out all converted terms from Eq. S13 and taking the square root, we find the dimensionless relationship between CV_Y_, spatial CV and the modifying effects of synchrony and persistence:

| $\text{CV}_{\text{Y}}=\left(\frac{\text{n}_{\text{k}}}{\text{n}_{\text{i}}}\widetilde{\text{CV}}_{\text{k}}^{2}+\frac{\text{n}_{\text{i}}}{\text{n}_{\text{i}}-1}\cdot\frac{\text{φ}_{\text{T}}\text{Var}\left(\text{Y}\right)_{\text{max}}-\sum^{ } \text{var}\left(\text{X}_{\text{i}}\right)}{\left(\overline{\text{X}_{\text{Y}}}\right)^{2}}-\frac{\text{n}_{\text{i}}}{\text{n}_{\text{k}}^{2}-\text{n}_{\text{k}}}\cdot\frac{\text{φ}_{\text{S}}\text{Var}\left(\text{Z}\right)_{\text{max}}-\sum^{ } \text{var}\left(\text{X}_{\text{k}}\right)+2\left(\left(\text{n}_{\text{k}}-1\right)\sum^{ } \text{cov}\left(\text{X}_{\text{k}},\text{X}_{\text{l}}\right)_{\text{max}}\right)}{\left(\overline{\text{X}_{\text{Y}}}\right)^{2}}\right)^{\frac{1}{2}}$ |  | Eq. S28 |
| --- | --- | --- |

While φ_T_ and φ_S_ do not contribute in a simple way to CV_Y_, these indices of synchrony and persistence have the same effect on temporal variability as expected from Eq. S13: All else equal, an increase in φ_T_ increases CV_Y_, while increasing φ_S_ decreases CV_Y_.

3. Analytical approximation of regional temporal CV value for “independent dynamics”

Eq. S28 presents regional (aggregate) temporal CV in terms of a weighted-mean spatial CV and indices of synchrony (φ_T_) and persistence (φ_S_). But when φ_T_ and φ_S_ are zero, their terms do not drop out fully, making it difficult to derive a null expectation. Furthermore, φ_T_ and φ_S_ values of zero actually reflect negative covariance among patches and among time points, respectively. In contrast, we are interested in the case of independent dynamics, where inter-patch and inter-time covariances are zero. We therefore derive a simpler approximation of regional temporal CV values that applies to zero covariances, and will be more useful for generating expected CV’s.

From [5], we note that when patches are uncorrelated in time, regional temporal CV is proportional to the mean temporal CV of patch *i* as a function of patch number *n_i_*:

| $\text{CV}_{\text{Y}}=\text{ }\frac{1}{\sqrt{\text{n}_{\text{i}}}}\overline{\text{CV}_{\text{i}}}$ |  | Eq. S29 |
| --- | --- | --- |

When values are uncorrelated among time points (i.e., low persistence) in addition to among patches, ergodicity applies. This means that, on average, the temporal CV of patch *i* will be roughly equivalent to the spatial CV at time *k*:

| $\overline{\text{CV}_{\text{i}}}\cong\text{ }\overline{\text{CV}_{\text{k}}}$ |  | Eq. S30 |
| --- | --- | --- |

Substituting Eq. S30 into S29, we find it possible to estimate regional CV values (CV_Y_) from spatial CV’s (CV_k_) when dynamics are independent in time and space:

| $\text{CV}_{\text{Y}}\cong\text{ }\frac{1}{\sqrt{\text{n}_{\text{i}}}}\overline{\text{CV}_{\text{k}}}$ |  | Eq. S31 |
| --- | --- | --- |

The CV_Y_ approximation compared well with values drawn from random datasets designed to simulate independent dynamics (see below). Values from the two methods were 94 and 98% correlated when using arithmetic and logged values, respectively. Regressions had slopes near one and y-intercepts near zero.

4. Robustness and behavior of the spatial-temporal variability relationship in a null model

We verified the observed relationship between spatial and temporal variability, and explored the conditions of its emergence, using a stochastic null model. Random numbers, corresponding to the range of microcosm experiment values, were drawn from a uniform distribution in Excel 14.0 (Microsoft Corporation 2010) and were assigned to arrays of three patches over 20 time steps. Spatial CV and regional temporal CV were estimated in replicate arrays of patches for 20 null “variables” representing processes with the same statistical properties. These variables were allowed to randomly vary within the same bounds (i.e., 0.001-28.000).

No regressions of mean spatial CV and temporal CV were significant (Fig. 3B; r^2^ = 0.06, p = 0.28, n = 20), demonstrating that intrinsic properties of variables, and not statistical happenstance, generate empirical patterns. This result also suggests that relationships will be undetectable in real variables when they share similar variation around the mean in space and/or in time. Cases of similar variability that might obscure relationships include; measurement error, measurements of the same variable in replicate systems, species that vary over similar spatiotemporal scales (see scatter around biotic variables in Fig. 4B) or any other variables with similar rates of spatial and temporal change (e.g., chlorophyll a, phytoplankton biomass and primary productivity [6]). In contrast, significant regressions emerged when some variables differed meaningfully from others in their spatial and temporal CV’s. Variability of a variable was manipulated by constraining patch values to a randomly chosen range, and assigning a different range (i.e., level of constraint) to each variable. The result was variables which span a range of spatial and temporal CV’s to form a (positive) near-unity relationship on a log-log plot (Fig. 3C-D).

Our null model also illustrated how the positions of points on a spatial-temporal CV plot change with the degree of synchrony or persistence experienced by a variable. Moreover, these responses agreed entirely with expectations from Fig. 1 and Eqs. S12 and S28. For a single variable (point), increasing synchrony among patches caused data points to migrate above the regression line, while increasing persistence moved the point below the line. This pattern held for the collective of variables too: When synchrony was induced among patches systematically for all variables, the regression slope remained the same, but all points shifted upwards on the plot, increasing the y-intercept (Fig. 3C). Enhancing persistence, in contrast, displaced all data points downwards, decreasing the y-intercept (Fig. 3C). When a variable’s spatiotemporal pattern was a function of its variability, slopes deviated from 1 as shown by Fig. 3D.

Extreme behaviors occur in spatial-temporal CV plots when (i) patches have identical means and are perfectly synchronized or (ii) patches have different means and show perfect persistence. In these cases, data points of the slope approach: (i) a vertical line for perfect synchrony because temporal variability exists but spatial variability is zero for all variables, and (ii) a horizontal line for perfect persistence because spatial variability exists but temporal variability is zero in all variables. It is important to note, however, that natural systems do not experience these extremes of order.

5. Supplementary materials and methods

Variables in the aquatic microcosm experiment were measured using the protocols listed in Table S1. Table S2 lists variables from the three datasets included in analyses.

6. Alternative estimators of variability

Choices exist for indices to estimate the three components of temporal variability (Eqs. S12, S28). The Coefficient of Variation (CV) is a common estimator of variability in space and time. However, the CV does not always remove the effect of the mean, and may produce biased estimates of variability [10,11]. We guarded against this possibility by reanalyzing data with a range of alternative variability indices (Table S3). Trends were detectable using all indices, and proved insignificant in only two cases. One case used the standard deviation of the logarithms, SD[Log(x)][12], and one the Population Variability [13] index. Insignificant results, representing four percent of tests, were likely due to the low statistical power of those particular analyses. In all cases, however, semi-partial correlations between temporal variability and spatial variability, synchrony or persistence were of the same sign predicted by our analytical relationship. Results from a CV-based null model (Fig. 3) were also reproducible using alternative indices, further suggesting robustness of the relationship.

Fewer scale-free indices estimate synchrony or persistence. We estimated these using spatial and temporal variance ratios of the form φ_x_ [3] because their common alternative, the mean pairwise (Pearson) correlation among patch time series or spatial series [14], cannot accommodate rows or columns of only zeroes. This shortcoming comes about because correlation between a row/column of zeroes and another row/column is undefined. If undefined correlations are excluded, the metric is calculated from fewer *n*. This, in turn, introduces a bias towards lower synchrony estimates [3]. Nonetheless, using the correlation-based indices yielded qualitatively similar - but not identical - results in almost all analyses.

7. Spatial imprinting of temporal processes

A space-time correspondence has long been recognized by ecologists, for instance in the tandem increase in a process’s spatial scale with its temporal scale [16,17]. We presented and tested an exact formulation of an underlying relationship between spatial and temporal variation. Below, we sketch the conceptual links between these dimensions of variation. As a starting point, regional variation over time of a population or a process, in a landscape of local patches (*i*…*n*), can be thought of as the sum of patch temporal variances and covariances among patches (Eq. S9). Regional variation thus arises from two temporal sources; localized, patch-scale changes over time (∑var(X_i_)) and fluctuations that are shared among those patches (∑cov(X*_i_,*X*_j_*)). Figs. 1 and 2 in the main text sketch how the variation and covariation of patches can be seen through a spatial lens; as a series of spatial snapshots, each with a distinct landscape pattern.

A spatial snapshot reflects both the variation of individual patches and covariation among them. When this covariation is minimal, the snapshot of spatial variation can be attributed solely to the temporal variation of individual patches. Spatial variation thus scales with, and becomes an index for, the typical magnitude of temporal variation (Fig. 2).

Many ecological mechanisms alter the temporal variation of local patches (∑var(X_i_)), and so should affect spatial variation also. Stabilizing mechanisms (Table S4) may be common in nature, but little consideration has been given to their influence on spatial variation. For instance, dispersal or forcing from extrinsic sources can stabilize dynamics within patches by dampening amplitudes of fluctuations. In doing so, stabilizing mechanisms should reduce the likelihood that any patch has a larger population than another and thus dampen spatial variability. It must be noted, however, that differences among patches will not be entirely erased if patches stabilize to different population sizes (e.g., have different equilibria) and stable patches may still create high spatial variation. We term this situation *persistence of spatial variation* (also known as *fixed spatial variation* [18]) and point out that when it occurs, spatial and temporal variation share a different relationship. Thus, the meaning of a spatial snapshot changes depending on how much persistence is displayed by a variable.

Because a spatial snapshot also captures inter-patch synchrony, the flows of individuals, energy or particles among patches and shared exposure to environmental factors like weather [19] take on considerable importance. By redistributing energy and matter, these phenomena impose simultaneous changes to spatial and temporal patterns. For instance, it is well-known that dispersal or environmental forcing can synchronize patches (increase ∑cov(X*_i_,*X*_j_*)) which is destabilizing at the regional scale [20](Fig. 5). But this also minimizes spatial differences, lowering the variability of patch densities across the landscape by making fluctuations coincide. Therefore, synchrony - like persistence - modifies the interpretation of what a spatial snapshot means for the temporal variation of a process.

**Supplementary references**

1. Sokal RR, Rohlf FJ (1981) Biometry. 2nd ed. New York: W. H. Freeman and Co.

2. Feller W (1950) An introduction to probability theory and its applications. New York: Wiley.

3. Loreau M, De Mazancourt C (2008) Species synchrony and its drivers: Neutral and nonneutral community dynamics in fluctuating environments. The American Naturalist 172: E48–E66.

4. Thibaut LM, Connolly SR (2013) Understanding diversity-stability relationships: Towards a unified model of portfolio effects. Ecology letters 16: 140–150.

5. Tilman D, Lehman CL, Bristow CE (1998) Diversity-stability relationships: Statistical inevitability or ecological consequence? The American Naturalist 151: 277–282.

6. Rocha MR, Vasseur DA, Hayn M, Holschneider M, Gaedke U (2011) Variability patterns differ between standing stock and process rates. Oikos 120: 17–25.

7. Wetzel RG, Likens GE (1991) Limnological analyses. 2nd ed. New York: Springer-Verlag.

8. Ritchie RJ (2006) Consistent sets of spectrophotometric chlorophyll equations for acetone, methanol and ethanol solvents. Photosynthesis Research 89: 27–41.

9. Warren CR (2008) Rapid measurement of chlorophylls with a microplate reader. Journal of Plant Nutrition 31: 1321–1332.

10. Gaston KJ, Mcardle BH (1994) The temporal variability of animal abundances: Measures, methods and patterns. Philosophical Transactions of the Royal Society of London B 345: 335–358.

11. Cottingham KL, Brown BL, Lennon JT (2001) Biodiversity may regulate the temporal variability of ecological systems. Ecology Letters 4: 72–85.

12. Lewontin RC (1966) On the measurement of relative variability. Systematic Zoology 15: 141–142.

13. Heath JP (2006) Quantifying temporal variability in population abundances. Oikos 115: 573–581.

14. Liebhold A, Koenig WD, Bjørnstad ON (2004) Spatial synchrony in population dynamics. Annual Review of Ecology, Evolution and Systematics 35: 467–490.

15. Downing JA (1991) Biological heterogeneity in aquatic ecosystems. In: Kolasa J, Pickett STA, editors. Ecological Heterogeneity. New York: Springer-Verlag. p. 332.

16. Holling CS (1992) Cross-scale morphology, geometry, and dynamics of ecosystems. Ecological Monographs 62: 447–502.

17. Steele JH, Henderson EW, Mangel M, Clark C (1994) Coupling between physical and biological scales [and discussion]. Philosophical Transactions of the Royal Society of London B 343: 5–9.

18. Lewis WM (1978) Comparison of temporal and spatial variation in the zooplankton of a lake by means of variance components. Ecology 59: 666–671.

19. Paradis E, Baillie R, Sutherland WJ, Gregory RD (1999) Dispersal and spatial scale affect synchrony in spatial population dynamics. Ecology Letters 2: 114–120.

20. Earn DJD, Levin SA, Rohani P (2000) Coherence and conservation. Science 290: 1360–1364.

21. Abbott K (2011) A dispersal-induced paradox: Synchrony and stability in stochastic metapopulations. Ecology Letters 14: 1158–1169.

22. Gonzalez A, Lawton JH, Gilbert FS, Blackburn TM, Evans-Freke I (1998) Metapopulation dynamics, abundance, and distribution in a microecosystem. Science 281: 2045–2047.

23. Lecomte J, Boudjemadi K, Sarrazin F, Cally K, Clobert J (2004) Connectivity and homogenisation of population sizes: An experimental approach in Lacerta vivipara. Journal of Animal Ecology 73: 179–189.

24. Amarasekare P (2004) The role of density-dependent dispersal in source-sink dynamics. Journal of Theoretical Biology 226: 159–168.

25. Leavitt PR, Fritz SC, Anderson NJ, Baker PA, Blenckner T, et al. (2009) Paleolimnological evidence of the effects on lakes of energy and mass transfer from climate and humans. Limnology and Oceanography 54: 2330–2348.

26. Kling GW, Kipphut GW, Miller MM, O’Brien WJ (2000) Integration of lakes and streams in a landscape perspective: The importance of material processing on spatial patterns and temporal coherence. Freshwater Biology 43: 477–497.

27. With KA, Schrott GR, King AW (2006) The implications of metalandscape connectivity for population viability in migratory songbirds. Landscape Ecology 21: 157–167.

28. Coulson T, Guiness F, Pemberton J, Clutton-Brock T (2004) The demographic consequences of releasing a population of Red Deer from culling. Ecology 85: 411–422.

29. Vasseur DA, Fox JW (2007) Environmental fluctuations can stabilize food web dynamics by increasing synchrony. Ecology Letters 10: 1066–1074.
