# Supplementary material for: Spatial Variation as a Tool for Inferring Temporal Variation and Diagnosing Types of Mechanisms in Ecosystems
Source: PLoS One. 2014 Feb 20;9(2):e89245. doi: 10.1371/journal.pone.0089245 (PMC3930753; doi:10.1371/journal.pone.0089245)
Supplement: Table S1 — Community and ecosystem variables measured over the 20-week microcosm connectivity experiment. (DOCX) [file pone.0089245.s002.docx]

**Table S1.** Community and ecosystem variables measured over the 20-week microcosm connectivity experiment

| **Variable** | **Measurement / method** |
| --- | --- |
| Total ecosystem respiration | Rate of dissolved oxygen loss in a ‘dark’ bottle of microcosm water. A homogeneous sample of the community within a microcosm was incubated *in situ* in a sealed, lightproof, 25 mL glass vial for three hours, always at the same time of day [7]. Precision: ±3% |
| Net primary productivity | Rate of dissolved oxygen gain in a 25 mL ‘light’ bottle of microcosm water. The remaining procedures followed those applied to ‘dark’ bottles [7]. Precision: ±3% |
| Gross primary productivity | Combined absolute magnitudes of the above two measurements [7]. Precision: ±6% |
| Phytoplankton (>1μm) density | Spectral absorption of chlorophyll-a on a BioTek Synergy 2 microplate reader (Winooski, Vermont, USA). A 12 mL sample of homogenized microcosm water was concentrated on a 1.1 µm glass micro-fiber filter, boiled in 2.5 mL of 95% ethanol for 20 minutes and extracted for 24 hours [8,9]. Precision: ±3% |
| Picoplankton / particulate (<1μm) density | Spectral absorption at 550 nm of 2 mL of microcosm water after being passed through a 1.1 µm glass micro-fiber filter. Precision: ±22% |
| Dissolved oxygen | Microcosm dissolved oxygen concentration, as measured by an ExStik^®^ DO600 probe (Waltham, Massachusetts, USA). Precision: ±0.4% |
| Temperature | Microcosm water temperature. Precision: ±0.3% |
