# Supplementary material for: Spatial Variation as a Tool for Inferring Temporal Variation and Diagnosing Types of Mechanisms in Ecosystems
Source: PLoS One. 2014 Feb 20;9(2):e89245. doi: 10.1371/journal.pone.0089245 (PMC3930753; doi:10.1371/journal.pone.0089245)
Supplement: Table S2 — Variables from lake, rock pool and microcosm data sets used in analyses. An additional 36 rock pool species (not shown), known from fewer occurrences, were included for calculating spatiotemporal signatures (Fig. 6). (DOCX) [file pone.0089245.s003.docx]

**Table S2.** Variables from lake, rock pool and microcosm data sets used in analyses. An additional 36 rock pool species (not shown), known from fewer occurrences, were included for calculating spatiotemporal signatures (Fig. 6).

| **Lakes LTER** | | **Rock pools** | **Microcosms** |
| --- | --- | --- | --- |
| Magnesium | *Chaoborus* larvae | Temperature | NPP |
| Sodium | *Chaoborus* pupae | Salinity | GPP |
| Sulfate | Leptodora | Dissolved oxygen | Ecosystem respiration |
| Calcium | Mysis | pH | Chlorophyll-a |
| Chloride | Rainbowsmelt | Oxygen saturation | Picoplankton/particulate |
| Potassium | Blackbullhead | Chlorophyll a | Dissolved oxygen |
| Iron | Blackchinshiner | Total invert. abundance | Temperature |
| Manganese | Blackcrappie | *Culex* sp. |  |
| Specific conductance | Blacknoseshiner | *Paracyclops* sp. |  |
| pH | Bluegill | *Orthocyclops* sp. |  |
| Alkalinity | Bluntnosedminnow | *Gyratrix hermaphroditus* |  |
| Dissolved inorganic carbon | Burbot | *Sesarma miersi* larvae |  |
| Total inorganic carbon | Commonshiner | Ceratopogonid sp. |  |
| Dissolved organic carbon | Fathead | *Candona* sp. |  |
| Total organic carbon | Goldenshiner | Oligochaeta sp. |  |
| Nitrate | Iowadarter | *Cyrpicercus* sp. |  |
| Ammonia | Johnnydarter | *Cytheromorpha* sp. |  |
| Total nitrogen (filtered) | Largemouthbass | *Cyrprinotus heterocypris* |  |
| Total nitrogen (unfiltered) | Logperch | *Nitocra* sp. |  |
| Total phosphorus (filtered) | Mimicshiner | *Alona davidii* |  |
| Total phosphorus | Mottledsculpin | Nematoda sp. |  |
| (unfiltered) | Mudminnow | *Cerodaphnia regaudi* |  |
| Dissolved reactive silica | Pumpkinseed | *Potamocyrpis* sp. |  |
| Bicarbonate reactive silica | Smallmouthbass | *Orthocyclops modestus* |  |
| Total organic matter | Walleye | *Cypridopsis mariae* |  |
| Temperature | Whitesucker | *Nitocra spinipes* |  |
| Dissolved oxygen | Yellowbullhead | *Leydigia* *leydigi* |  |
| O_2_ saturation | Yellowperch | *Metis* sp. |  |
| Surface light | Northernpike | *Cyprideis* sp. |  |
| Light at depth | Rockbass | Chronomid sp. |  |
| Light attenuation |  | *Cytheromorpha* sp. |  |
|  |  | Dorvelleidae sp. 1 |  |
|  |  | Dorvelleidae sp. 2 |  |
