# Supplementary material for: Spatial Variation as a Tool for Inferring Temporal Variation and Diagnosing Types of Mechanisms in Ecosystems
Source: PLoS One. 2014 Feb 20;9(2):e89245. doi: 10.1371/journal.pone.0089245 (PMC3930753; doi:10.1371/journal.pone.0089245)
Supplement: Table S3 — Comparison of CV-based results with alternative indices of variability. General Linear Models were fit between indices of aggregate temporal variability and three spatiotemporal descriptors: Spatial variability, inter-patch synchrony and persistence (see Fig. 1B). Indices of variability included CV and four others. Asterisks denote statistical significance and p-value. *p<0.05, **p<0.01, ***p<0.001. R2 values in parentheses. (DOCX) [file pone.0089245.s004.docx]

**Table S3.** Comparison of CV-based results with alternative indices of variability. General Linear Models were fit between indices of aggregate temporal variability and three spatiotemporal descriptors: Spatial variability, inter-patch synchrony and persistence (see Fig. 1). Indices of variability included CV and four others. Asterisks denote statistical significance and p-value. * p <0.05, ** p < 0.01, *** p < 0.001. R^2^ values in parentheses.

| **Index** | **Description** | **Significant relationship between temporal variability and:** | | |
| --- | --- | --- | --- | --- |
|  |  | Mean spatial variability? | Spatial variability + synchrony? | Spatial variability + synchrony + persistence? |
| **Coefficient of Variation (CV)** | Standard measure of relative variation. Unaffected by zeroes. May be biased by mean. | *Microcosms****^***^***(0.93) *Rock pools****^***^*** (0.77) *Lakes****^***^***(0.73) | *Microcosms****^***^***(0.98) *Rock pools****^***^*** (0.86) *Lakes****^***^***(0.80) | *Microcosms****^***^*** (1.00) *Rock pools****^***^*** (0.94) *Lakes****^***^***(0.87) |
| **SD [log(x)]**[12] | Variation of log-transformed values. Problematic for data with zeroes; not applied to most species data. | *Microcosms****^**^*** (0.81) *Rock pools****^**^***  (0.91) *Lakes****^**^*** (0.22) | *Microcosms****^*^*** (0.83) *Rock pools****^*^*** (0.92) *Lakes****^*^*** (0.25) | *Microcosms****^n.s.^*** (0.85) *Rock pools****^*^*** (0.99) *Lakes****^**^***(0.45) |
| **Bray-Curtis similarity** | Measures similarity of values in time or space series as ratio of value sums and differences. Robust to zeroes. | *Microcosms****^**^***(0.90) *Rock pools****^***^*** (0.58) *Lakes****^***^*** (0.76) | *Microcosms* ***^**^***(0.94) *Rock pools****^***^***(0.70) *Lakes****^***^***(0.77) | *Microcosms****^*^***(0.94) *Rock pools****^***^***(0.74) *Lakes****^***^***(0.84) |
| **Population Variability** [13] | Average percent difference among values. Species and abiotic variables analyzed separately. | *Microcosms****^***^***(0.96) *Rock pools****^**^***(0.35, 0.73) *Lakes****^**^***(0.07, 0.36) | *Microcosms****^***^***(0.98) *Rock pools****^**^***(0.44, 0.91) *Lakes****^**^***(0.22, 0.37) | *Microcosms****^**^***(0.98) *Rock pools****^**^***(0.50, 0.98) *Lakes****^***^***(0.61, 0.41) |
| **Downing’s B** [15] | Empirically-derived CV variant to remove effect of mean. Applied to invertebrate data only. | *Rock pools****^***^***(0.49) | *Rock pools****^***^***(0.57) | *Rock pools****^***^***(0.76) |
