# Supplementary material for: Spatial Variation as a Tool for Inferring Temporal Variation and Diagnosing Types of Mechanisms in Ecosystems
Source: PLoS One. 2014 Feb 20;9(2):e89245. doi: 10.1371/journal.pone.0089245 (PMC3930753; doi:10.1371/journal.pone.0089245)
Supplement: Table S4 — Mechanisms that may dampen regional variability and reduce spatial variability by stabilizing local patches. (DOCX) [file pone.0089245.s005.docx]

**Table S4.** Mechanisms that may dampen regional variability and reduce spatial variability by stabilizing local patches.

| **Type** | **Mechanism** | **Examples** |
| --- | --- | --- |
| Spatial exchange | Movement to or from patch that changes local regulation of dynamics and reduces size (amplitude) of fluctuations | Source-sink dynamics or “Rescue effects” that enhance population viability or limit overcompensation [21–24] |
| Spatial exchange | Effluxes to or influxes from outside system equilibrate across landscape and compensate for any local decreases or increases | Diffusive environmental fluxes [25,26]; mobile, wide-ranging organisms [27] |
| Exogenous forcing | Forcing by an exogenous variable over a large extent constrains rates of increase and variability of all patches | Demographic regulation by mobile predators [28] or by environmental forcing [29] |
